# FH-deficient uterine leiomyoma: A rare case report highlighting diagnostic and clinical challenges

**DOI:** 10.1016/j.radcr.2025.11.001

**Published:** 2025-12-04

**Authors:** Wissal Jaafar, Yasmine Chiba, Malak Medemagh, Souha Gabbouj, Mehdi Bouassida, Nahed Khalifa, Imen Ganzoui, Mechaal Mourali

**Affiliations:** a Department of Gynecology and Obstetrics, Bougatfa Hospital, Bizerte, Tunisia; b Department of Radiology, Habib Bougatfa Hospital, Bizerte, Tunisia

**Keywords:** FH-deficient leiomyoma, Fumarate hydratase deficiency, Myomectomy, Pelvic pain, Uterine leiomyoma

## Abstract

Fumarate hydratase (FH)–deficient uterine leiomyomas are a rare subtype of fibroids with distinctive histological and immunohistochemical features. We report the case of a 44-year-old nulligravida presenting with chronic pelvic pain for 2 years and an enlarged abdomen. Pelvic ultrasound revealed a polymyomatous uterus with the largest myoma measuring 14 cm, and MRI confirmed multiple leiomyomas with signs of pelvic congestion. The patient underwent polymyomectomy, and histopathological analysis identified an Fumarate hydratase (FH)-deficient leiomyoma. This entity is important to recognize given its potential association with Hereditary Leiomyomatosis and Renal Cell Carcinoma (HLRCC), warranting careful diagnosis, appropriate surgical management, and consideration of genetic counseling.

## Introduction

Uterine leiomyomas are the most frequent benign tumors of the female genital tract, usually diagnosed in women during their forties [1]. They are smooth muscle neoplasms with a broad spectrum of histological subtypes. Among them, fumarate hydratase (FH)–deficient leiomyomas represent a rare variant, reported in about 0.4%-1% of all cases [2].

These tumors often appear at a younger age and may occur either sporadically or in association with hereditary Leiomyomatosis and renal cell carcinoma (HLRCC), a rare autosomal dominant condition characterized by multiple cutaneous leiomyomas, symptomatic uterine fibroids, and a predisposition to highly aggressive renal carcinomas [3].

Accurate histopathological identification of FH-deficient leiomyomas is crucial, as it not only ensures correct diagnosis but also raises the need for further clinical and genetic evaluation. In this report, we describe the case of a 44-year-old woman diagnosed with an FH-deficient leiomyoma, underlining both the diagnostic challenges and the importance of recognizing potential genetic associations.

## Case report

We report, in accordance with the SCARE guidelines [4], the case of a 44-year-old nulligravida (G0P0) who presented with chronic pelvic pain persisting for 2 years. The patient had no notable past medical history and was not on any regular medication. There was no known family history of renal carcinoma, cutaneous leiomyomas, or other hereditary neoplastic conditions. The patient reported progressive abdominal distension without metrorrhagia. On physical examination, the abdomen was enlarged, with a firm, irregularly contoured mass palpable above the pelvic brim.

**Fig. 1:**
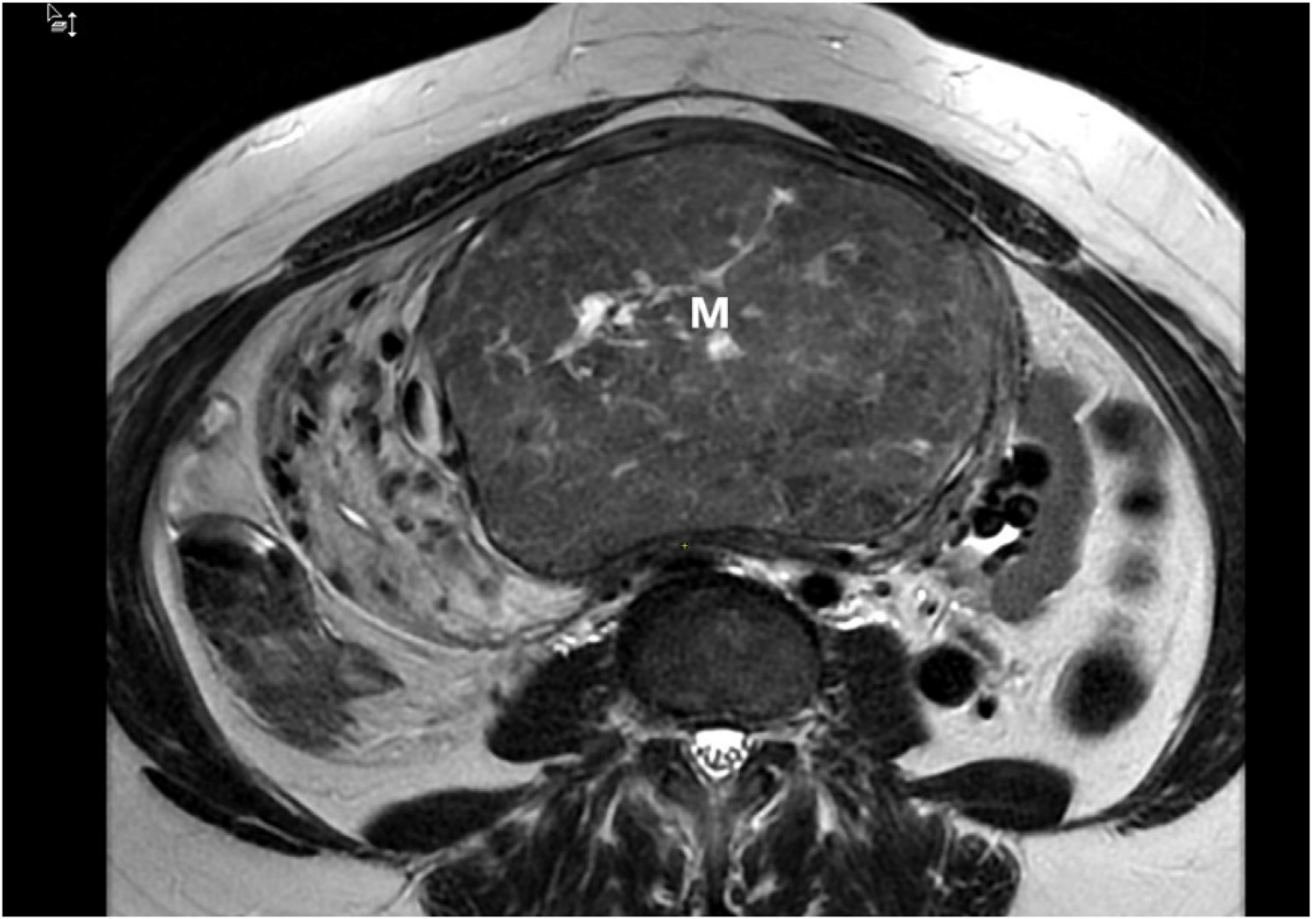
MRI image of the pelvis, axial image demonstrates a large heterogeneous exophytic mass arising from the uterus (M).

**Fig. 2:**
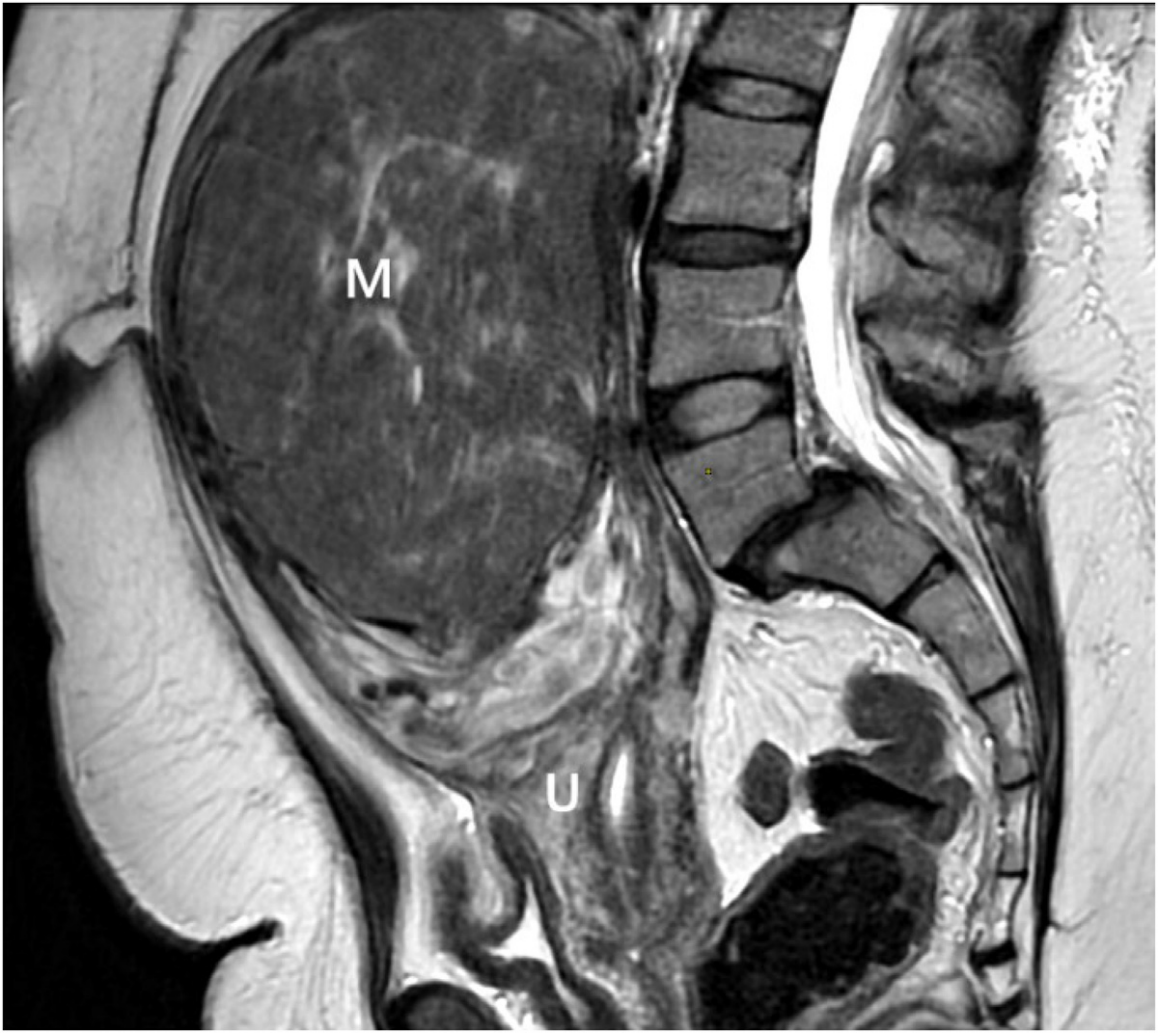
Sagittal T2 image demonstrates a large heterogeneous exophytic mass (M) arising from the uterus (U).

**Fig. 3:**
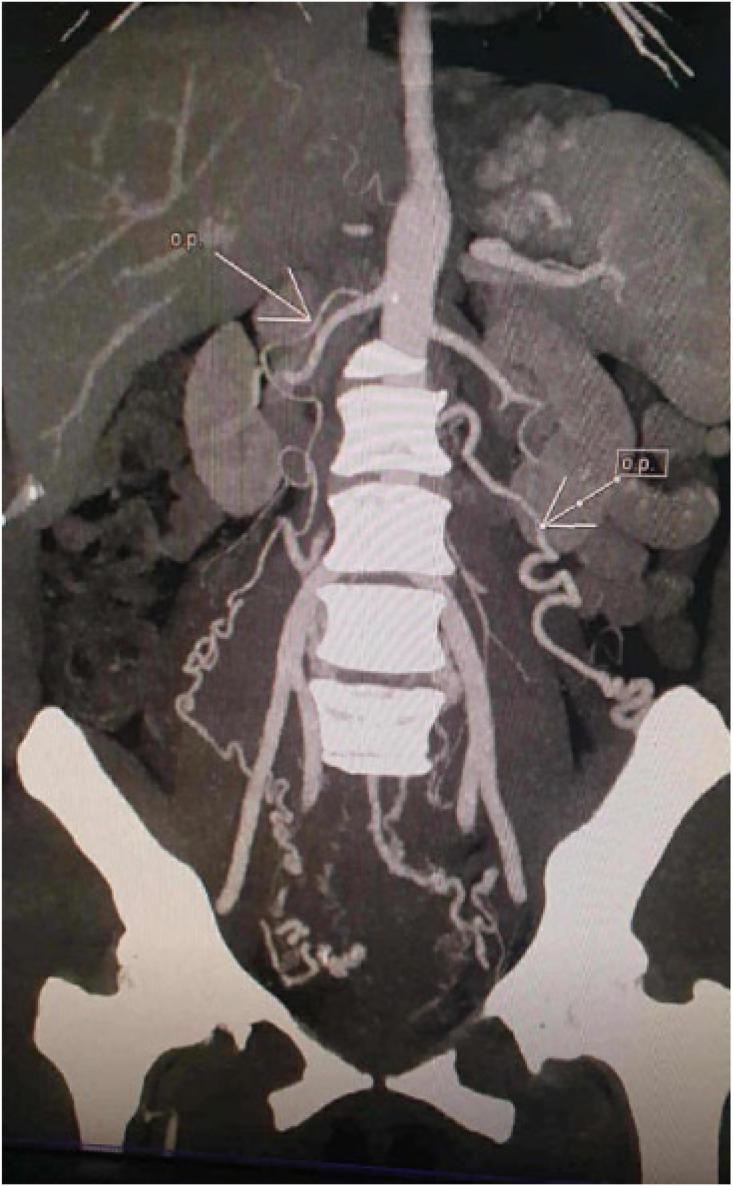
Contrast-enhanced CT image coronal image demonstrate a large heterogeneous mass with a pelvic venous reflux involving the left ovarian vein and the right internal iliac vein (Arrows).

Ultrasonography revealed a polymyomatous uterus with multiple intramural and subserosal fibroids of variable size. The largest myoma was located in the anterior uterine wall, appearing as a well-limited, hypoechoic, heterogeneous mass with posterior shadowing, and measuring approximately 14 cm in maximum diameter. Color Doppler study revealed increased vascularization of the mass. Pelvic MRI was performed to further characterize the lesions, which confirmed a markedly enlarged polymyomatous uterus. Three leiomyomas were identified, with the most voluminous lesion located anteriorly, classified as FIGO type 5, measuring 14 × 9 × 14 cm. This mass protruded into the abdominal cavity, exerting a mass effect on adjacent digestive structures, but without infiltration. On T2-weighted sequences, the lesion appeared heterogeneous, predominantly hypointense with areas of hyperintensity suggestive of edema and degeneration (Fig. 1, Fig. 2). After gadolinium injection, it showed heterogeneous enhancement with marked hypervascularization. Associated findings included a pelvic congestion syndrome, characterized by dilated myometrial and parametrial varices, as well as pelvic venous reflux involving the left ovarian vein and the right internal iliac vein on CT scan (Fig. 3).

In view of the severe symptomatology, uterine enlargement, and radiological features, the patient underwent polymyomectomy via laparotomy under general anesthesia. Intraoperatively, multiple intramural and subserosal leiomyomas were identified and excised completely, including the large anterior FIGO type 5 fibroid, with preservation of the uterine contour. Hemostasis was secured, and no intraoperative complications occurred. The postoperative course was uneventful, and the patient was discharged on the third postoperative day. At 6-month follow-up, clinical examination and pelvic ultrasound demonstrated normal uterine morphology with no recurrence or new myomatous lesions.

**Fig. 4:**
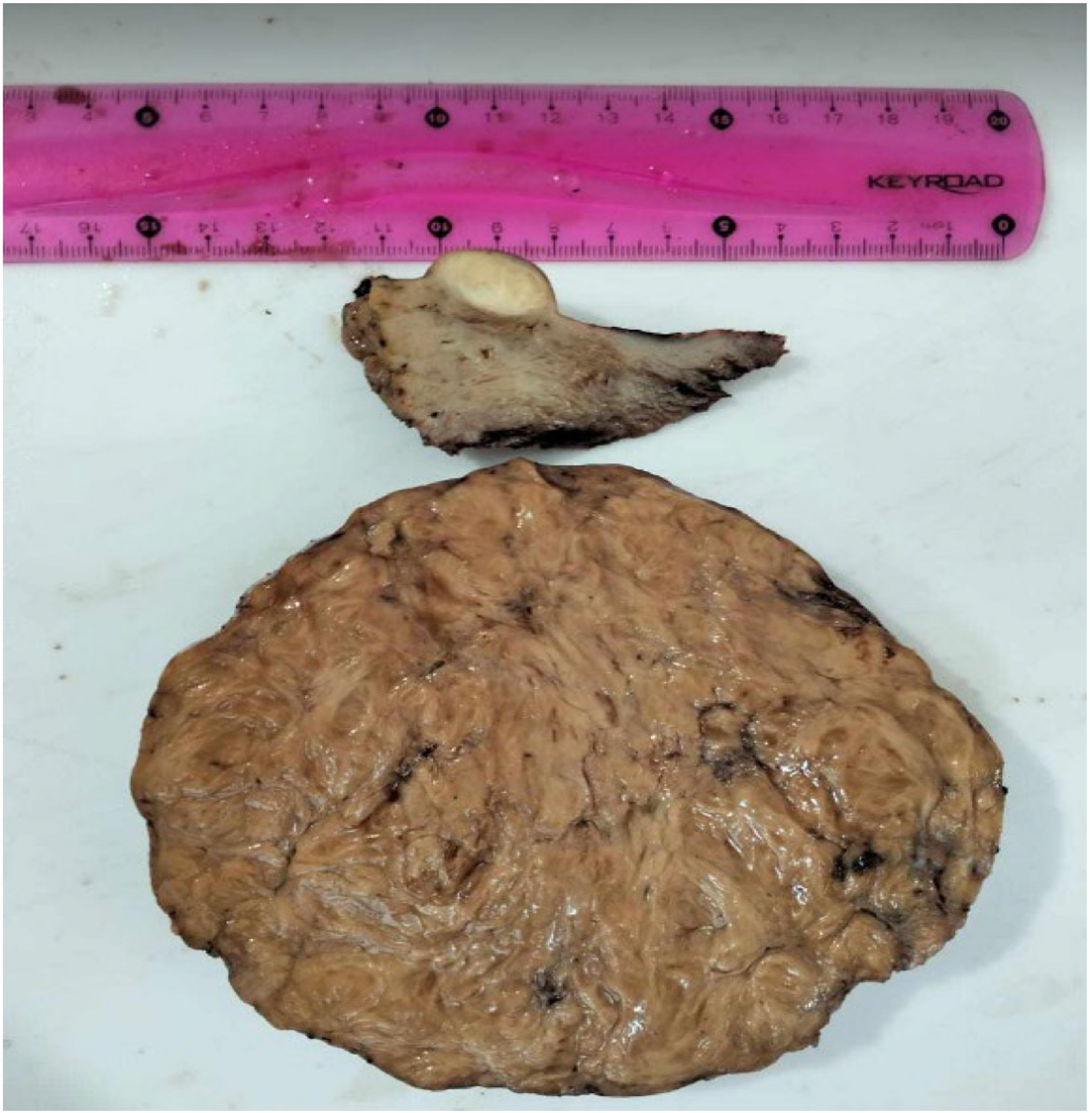
Myomectomy specimen.

Microscopic examination revealed a leiomyoma with atypical morphological features suggestive of FH deficiency . Immunohistochemistry confirmed the diagnosis by showing complete loss of FH expression, with positive staining for 2-succinocysteine (2SC) (Fig. 4).

Given the oncogenetic significance of this diagnosis and its potential association with HLRCC syndrome, the patient was referred for genetic counseling and multidisciplinary follow-up.

## Discussion

FH-deficient uterine leiomyomas are rare, accounting for approximately 0.4%-1% of all uterine leiomyomas in surgical series [2]. The true incidence is likely underestimated, as routine immunohistochemical screening for FH deficiency is not systematically performed. Most cases are *sporadic*, caused by somatic biallelic inactivation of the *FH* gene, whereas approximately *10%-20% occur in the context of HLRCC* syndrome due to germline *FH* mutations [5,6]. Recognition of this variant is therefore clinically important, as it may serve as a sentinel lesion prompting genetic counseling and renal surveillance.

Our case is distinguished by the presence of pelvic congestion syndrome associated with multiple hypervascular leiomyomas. Pelvic congestion results from dilated uterine and ovarian veins and venous reflux, and is a recognized cause of chronic pelvic pain in women [7,8]. We report this unique presentation, highlighting both the unusual molecular findings and the clinical significance of vascular complications in FH-deficient uterine leiomyomas.

On MRI, FH-deficient leiomyomas may present imaging features distinct from those of conventional uterine leiomyomas. Typically, they exhibit higher signal intensity on T2-weighted images due to cystic or edematous degeneration and may contain small areas of hemorrhage or necrosis. They often demonstrate heterogeneous enhancement after gadolinium administration, reflecting increased vascularity and cellular density. In contrast, typical leiomyomas usually appear as well-circumscribed, homogeneously hypointense masses on T2-weighted sequences with relatively uniform enhancement. These differences, although subtle, may raise suspicion of an FH-deficient subtype, particularly in younger patients or in cases showing atypical vascular patterns. However, definitive diagnosis still relies on histopathological and immunohistochemical confirmation of FH loss and 2-succinocysteine (2SC) positivity [7,8].

The FH gene, located on chromosome 1, encodes FH, present in both mitochondria and cytosol, which converts fumarate to L-malate in the TCA cycle. Germline mutations, whether heterozygous or homozygous, disrupt this conversion, leading to fumarate accumulation [5]. Heterozygous mutations predispose to FH-deficient leiomyomas and renal cell carcinoma, while homozygous mutations cause fumaric aciduria. Excess fumarate alters cellular metabolism, promoting anaerobic glycolysis, stabilizing hypoxia-inducible factor, and increasing oxidative stress. These changes contribute to DNA repair defects, angiogenesis, and oncogenic transformation [9].

FH-deficient leiomyomas exhibit distinctive microscopic characteristics that aid in their recognition. Histologically, they show intersecting fascicles of smooth muscle cells with cytologic atypia, including large nuclei, prominent eosinophilic nucleoli, and characteristic perinucleolar halos. The stroma may demonstrate alveolar-type edema and areas of hyalinization. Immunohistochemically, there is a complete loss of FH expression and a strong positive staining for 2-succinocysteine (2SC), which serves as a surrogate marker of FH deficiency.

The main differential diagnoses include *atypical (symplastic) leiomyoma* and *leiomyosarcoma*. Atypical leiomyomas display nuclear pleomorphism but lack the characteristic perinucleolar clearing and FH loss. Leiomyosarcomas, by contrast, show significant cytologic atypia, high mitotic activity, and areas of coagulative necrosis, features not present in FH-deficient leiomyomas. Therefore, the integration of histologic evaluation with immunohistochemical analysis is essential for accurate diagnosis and for identifying patients who may benefit from genetic counseling for HLRCC [1,7].

From a clinical perspective, the recognition of FH-deficient leiomyomas has major implications. First, their hypervascularity can increase intraoperative bleeding risk, making preoperative planning crucial.

Second, even in patients diagnosed at an older age, the detection of FH deficiency should prompt consideration of genetic counseling, given the non-negligible proportion of germline carriers and the associated risk of aggressive renal carcinoma in HLRCC. Finally, systematic follow-up with gynecologic and renal imaging is recommended in suspected hereditary cases to allow early detection of malignant transformation [5,6].

In summary, this case contributes to the limited literature by describing a rare presentation of FH-deficient leiomyoma associated with pelvic congestion syndrome, highlighting the importance of considering vascular complications in the clinical spectrum of this entity. Early recognition through histopathology and immunohistochemistry remains essential, not only for appropriate surgical management but also for genetic counseling and oncologic surveillance in the context of HLRCC.

## Conclusion

Awareness of FH-deficient uterine tumors is essential, as their recognition depends on familiarity with their distinctive morphological patterns and the systematic use of both FH and 2SC immunohistochemical markers. Maintaining a strong clinical suspicion can facilitate the identification of FH-deficient leiomyomas and, importantly, help uncover cases associated with HLRCC, enabling timely genetic evaluation and early management.

## Patient consent

A written and informed consent was obtained from patient and their attending family members before commencing the submission process.
